# Feasibility of prehospital delivery of remote ischemic conditioning by emergency medical services in chest pain patients: protocol for a pilot study

**DOI:** 10.1186/s40814-019-0431-8

**Published:** 2019-03-13

**Authors:** Mehul D. Patel, Timothy F. Platts-Mills, Joseph M. Grover, Sonia M. Thomas, Joseph S. Rossi

**Affiliations:** 1 Department of Emergency Medicine, University of North Carolina at Chapel Hill, 170 Manning Drive, CB #7594, Chapel Hill, NC 27599-7594 USA; 2 Orange County Emergency Services, Hillsborough, USA; 3 Division of Biostatistics and Epidemiology, RTI International, Raleigh, USA; 4 Division of Cardiology, University of North Carolina at Chapel Hill, Chapel Hill, USA

**Keywords:** Emergency medical services, Acute coronary syndrome, Chest pain, Ischemic conditioning, Feasibility study

## Abstract

**Background:**

Remote ischemic conditioning (RIC) is a non-invasive procedure with hypothesized therapeutic benefits for patients experiencing an acute ST-elevation myocardial infarction (STEMI). Further study of emergency medical services (EMS) delivery of RIC in the prehospital setting is needed to inform the design and methods for future clinical trials of RIC in STEMI patients. The main objective of this pilot study is to assess the feasibility of prehospital delivery of RIC by EMS providers in the United States.

**Methods:**

We will conduct a single-arm study of the standard RIC procedure (i.e., up to 4 cycles of alternating 5-min inflation and 5-min deflation of an upper arm cuff) administered by EMS paramedics in 50 patients experiencing acute onset chest pain. The investigational autoRIC® device (CellAegis Devices, Inc., Toronto, Ontario) will be initiated by paramedics during ground ambulance transport. Automated RIC cycles will continue through emergency department arrival and stay. The primary endpoint will be the completion of all 4 cycles of RIC without interruption. We will also examine study procedures and collect qualitative data from study participants and paramedics.

**Discussion:**

To our knowledge, this will be the first study in the United States to assess the feasibility of completing the 40-min RIC procedure when initiated during ground ambulance transport. Findings from this pilot study will be used to optimize the design and methods for a future efficacy trial of RIC in acute STEMI patients.

**Trial registration:**

NCT03400579 (ClinicalTrials.gov). Registered on 17 January 2018.

**Electronic supplementary material:**

The online version of this article (10.1186/s40814-019-0431-8) contains supplementary material, which is available to authorized users.

## Background

Coronary artery disease is a leading cause of mortality worldwide, with an estimated 7.4 million deaths in 2015 [1]. An acute ST-elevation myocardial infarction (STEMI), a very serious heart attack, occurs with a blockage of a major coronary artery and is typically characterized by symptoms of pain or pressure in the chest; pain radiating to the back, neck, jaw, and arms; shortness of breath; or nausea. If blood flow is not restored (reperfusion) within the first few hours, the lack of oxygen supply (ischemia) results in irreversible myocardial injury and eventually necrosis of heart tissue (infarction). Depending on the extent of tissue damage or myocardial infarct size, the individual may die or suffer disabling conditions like heart failure. While advancements in acute STEMI care have reduced mortality rates, chronic heart failure due to MI is becoming more prevalent and currently affects up to 30 million people globally [2].

Timely emergent percutaneous coronary intervention (PCI) is the recommended mode of reperfusion for acute STEMI [3]. Prompt reperfusion with primary PCI substantially reduces myocardial injury due to ischemia and improves clinical outcomes [3, 4]. However, reperfusion, as a result of restored blood flow, can paradoxically induce further myocardial damage [5]. This reperfusion injury likely involves several different mechanisms including oxidative stress, rapid pH changes, and mitochondrial dysfunction and can account for up to 50% of the final myocardial infarct size [6, 7]. While there is substantial evidence supporting reperfusion by PCI for STEMI, therapies to protect against reperfusion injury following PCI remain investigational and unproven [8].

Remote ischemic conditioning (RIC) is a non-pharmacological and non-invasive procedure whereby alternating brief episodes of benign ischemia and reperfusion are induced by inflating and deflating a standard cuff on the upper arm or leg [9, 10]. When administered prior to reperfusion, RIC is hypothesized to protect against reperfusion injury by cellular signaling between the remote site (limb) and the target organ (heart) through humoral or neuronal protective signal transfer [9, 11]. Although the mechanisms of RIC are unclear, it is thought to target several mediators of reperfusion injury and may, therefore, be a more effective therapeutic strategy than those that target a single pathway [6, 12]. There is substantial preclinical evidence from animal experiments demonstrating reduction of myocardial infarct size by RIC [13–15]. Several small, proof-of-concept clinical studies have shown that RIC, typically administered as 3 or 4 cycles of 5-min inflation and 5-min deflation of an upper arm cuff, safely improves cardiac biomarkers and left ventricular ejection fraction [9, 11]. Clinical trials have shown mixed findings with respect to RIC across different patient populations. For example, two large, multi-center trials recently failed to show a benefit of RIC on clinical outcomes in patients undergoing elective coronary artery bypass graft (CABG) surgery [16, 17] while a recent meta-analysis of four trials showed less major adverse cardiovascular events in STEMI patients receiving RIC prior to PCI [18]. Additional full-scale, high-quality trials are needed on the long-term clinical benefits of RIC in acute STEMI patients undergoing emergent PCI.

Emergency medical services (EMS) play a key role in regional systems of STEMI care in the United States (U.S.) and other developed countries and are a promising setting for field administration of RIC in acute STEMI patients prior to hospital arrival. Botker et al. conducted a randomized trial of RIC administered in the prehospital setting among 333 STEMI patients in Denmark, in which they found RIC was feasible to implement in the ambulance and safely improved myocardial salvage and reduced long-term major adverse cardiovascular events (hazard ratio = 0.49, 95% confidence interval = 0.27–0.89) [19, 20]. These researchers are currently conducting a large prehospital trial of RIC in four European countries in which RIC will be delivered during ambulance transport when feasible (NCT01857414) [21]. Further U.S.-based emergency care trials are needed to establish the efficacy and safety of RIC in a variety of EMS and acute STEMI care systems.

Given logistical challenges of prehospital emergency care research, pilot studies are required to inform the design and methods of future clinical trials of EMS-administered RIC in the U.S. As previously mentioned, European researchers have implemented RIC during ambulance transport with success. There are however important differences in the provision of EMS between European countries like Denmark, and the U.S. prehospital emergency care in European countries is often provided by emergency physicians and has a culture of treating patients at the scene whereas ambulances in the U.S. are staffed with paramedics who are trained to prioritize timely transport to the hospital [22, 23]. A recent feasibility study of administering RIC in patients with STEMI during U.S. air medical transports showed 84% of patients had at least 3 cycles of RIC completed [24]. In this study, the air medical crew was typically a paramedic and nurse team, which made timed, manual cuff inflations and deflations feasible. However, the vast majority of acute MI patients in the U.S. are transported by ground ambulance, not by air, with care provided by a single paramedic. Further study of RIC delivered by EMS during ambulance transport is needed to inform future prehospital trials.

### Objectives

The overall purpose of this pilot study is to assess the feasibility of prehospital delivery of RIC by paramedics in a U.S. EMS system. Our primary objective is to examine the duration of the RIC procedure administered in acute chest pain patients with an automated device initiated during ambulance transport. Since an automated device eliminates the need for dedicated personnel to perform manual cuff inflations [10, 25, 26] and based on a prior feasibility study [24], we hypothesize 4 cycles of RIC will be completed in at least 80% of patients having the procedure initiated. Further, we feel 80% is a reasonable lower bound to optimal RIC completion for a full-scale trial. Secondary objectives are to evaluate recruitment rates, implement the study intervention, assess acceptability of the study protocol by paramedics, and describe patient-reported experiences with the RIC procedure.

## Methods

### Study design and setting

**Fig. 1:**
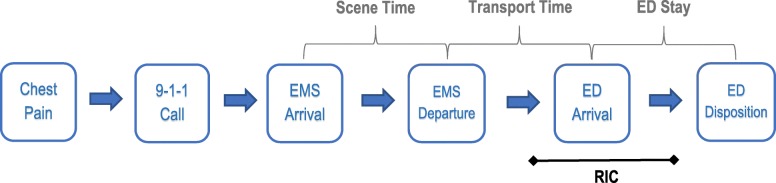
Overall process flow for eligible EMS response

This single-site, single-arm pilot study will recruit patients experiencing acute onset chest pain or anginal equivalent symptom who call 9-1-1 and are responded to by a ground ambulance crew. The study will be conducted in Orange County, North Carolina in the United States in partnership with Orange County Emergency Services, the sole advanced life support care provider in the county. Over a 15-month period, paramedics will identify eligible patients from the region of the county that predominantly transports to the emergency department (ED) of the University of North Carolina Medical Center (Chapel Hill, North Carolina), a 929-bed tertiary care academic hospital and accredited chest pain center. Figure 1 illustrates the general steps of an eligible EMS response.

### Study population

Eligible patients will be at least 18 years of age; require a 9-1-1 response to scene for non-traumatic chest pain or symptom suggesting cardiac etiology such as pain in other location, dyspnea, diaphoresis, nausea/vomiting, or dizziness; present within 12 h of symptom onset; and have a systolic blood pressure (SBP) between 100 and 180 mmHg. With the primary objective to evaluate the completion of 4 cycles of RIC in EMS transports, we defined a higher volume, lower risk study population to estimate this outcome within a practical scope and time period. Notably, patients meeting criteria for suspected STEMI based on field interpretation of a prehospital 12-lead electrocardiogram (ECG) will be excluded because these patients are taken directly to the cardiac catheterization laboratory and are not available for additional data collection in the ED. At the discretion of the paramedic, patients will be excluded if they have a pre-existing condition that precludes the administration of RIC, including paresis of the upper limb, traumatic injury to the arm, presence of an arteriovenous shunt for dialysis, prior mastectomy, existing peripheral inserted central catheter line, and arm edema or other indication of upper extremity thrombosis. Patients who are unconscious or otherwise in critical condition, lacking capacity to consent to the study, and do not speak English will also be excluded.

### Sample size

**Fig. 2:**
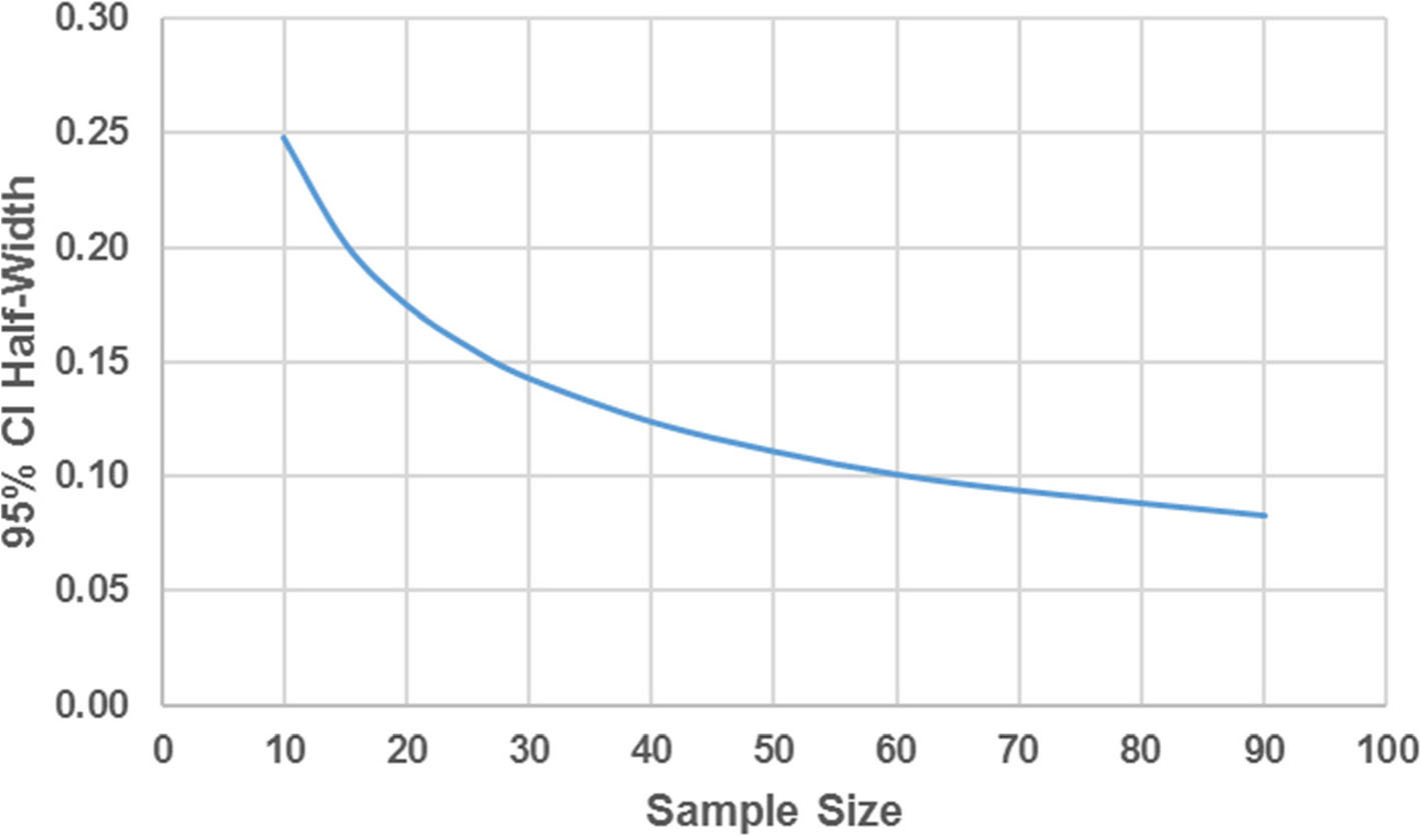
Statistical precision in the primary outcome by sample size

Since the primary objective of this pilot study is to assess feasibility and optimize methods, no formal statistical hypothesis testing will be performed. A target enrollment of 50 participants is reasonable within the 15-month pilot study period. This sample size would produce a 95% confidence interval (CI) half width of 0.11 for the proportion of participants with RIC completion given the proportion with RIC completion is 0.80 (primary hypothesis). Sample sizes larger than 50 provide only marginal gains in precision (Fig. 2).

### Intervention

In this pilot study, participants will receive RIC with the autoRIC® (CellAegis Devices, Inc., Toronto, Ontario)—a medical device designed specifically to administer 4 cycles of RIC. Once placed on the upper arm and initiated, the programmed device inflates the cuff to 200 mmHg and remains inflated for 5 min, after which the cuff is deflated and stays deflated for another 5 min. Each RIC cycle lasts 10 min, and 4 cycles require a total of 40-min intervention period. This RIC intervention is the standard intervention protocol used by completed and ongoing clinical trials. The autoRIC® has CE Mark certification in Europe and Health Canada approval to administer RIC therapy in adult patients with acute MI and those undergoing cardiothoracic interventions or surgery; the device is however limited to investigational use in the United States.

### Screening and recruitment

Three full-time ground ambulance units will be used for this study due to the limited number of autoRIC® devices. Paramedics assigned to these units will be responsible for assessing and screening patients during the enrollment period from 6 AM to 6 PM, Monday–Friday. So that arrival to the ED is not delayed, screening and recruitment will not begin until the patient is en route to the hospital and given usual care, at the discretion of the paramedic. Paramedics will assess the patients’ capacity to consent for the study following their usual approach to assessing patients’ capacity to make medical decisions, e.g., accept or refuse treatment.

**Fig. 3:**
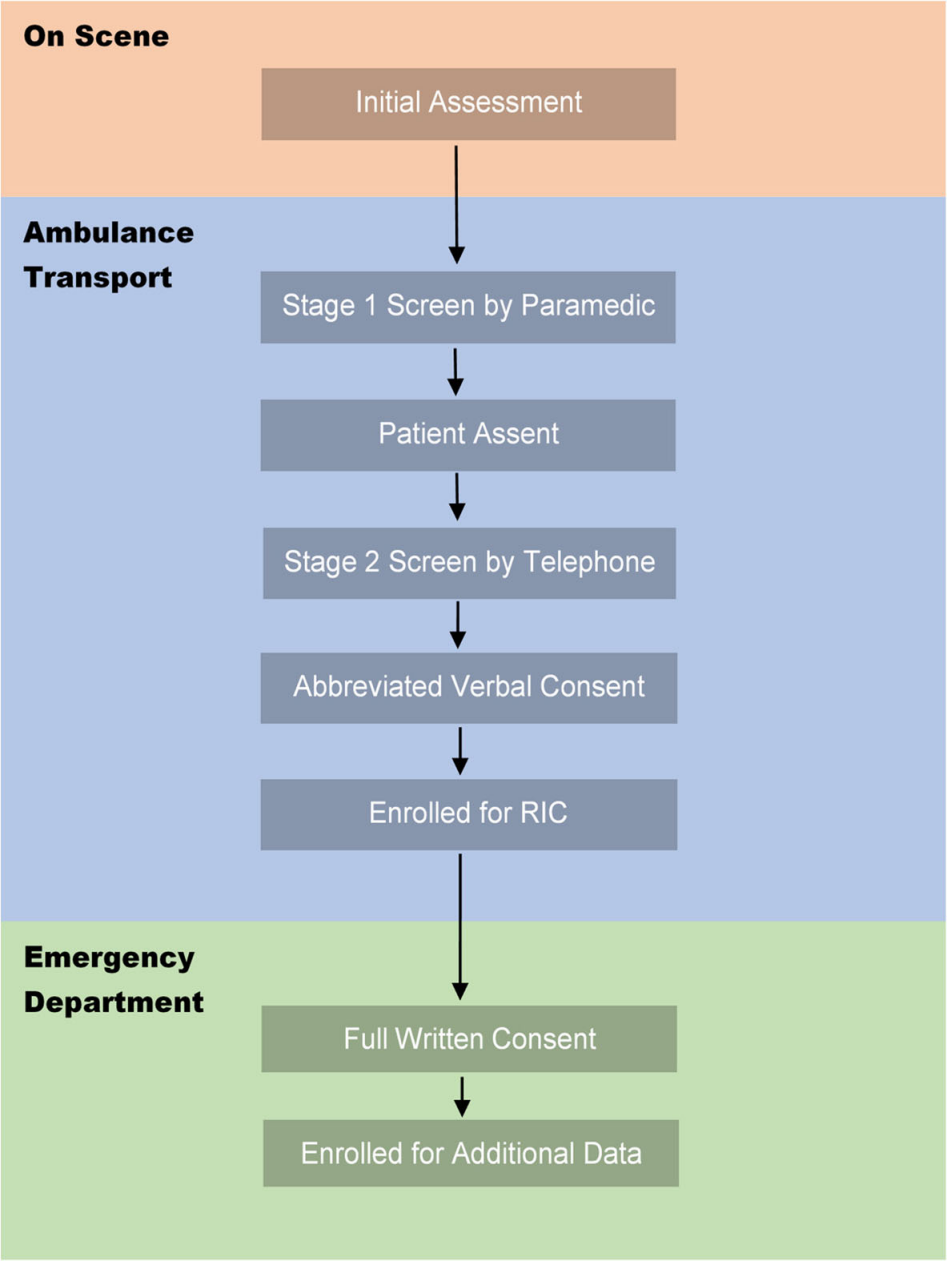
Flow chart of study recruitment and enrollment

In a two-stage screening and recruitment process (Fig. 3), the study paramedic will first assess and determine whether the patient meets all eligibility criteria. If the patient is not eligible, the paramedic will document the primary reason(s) on a screener form. If the patient is eligible, the paramedic, using a suggested script, will offer the patient an opportunity to learn more about the study by telephone call with a research assistant (RA). If the patient agrees, the paramedic will hand the patient a study information sheet and will call the RA or back-up study personnel using a dedicated cellular telephone. In the second screening stage, the RA will confirm with the paramedic that the patient is eligible. Then the patient will be handed the telephone. The RA, following a suggested script, will provide basic information about the purpose of the study, RIC procedure, and risks and benefits of participating and will answer any questions that the patient may have. To assess consent capacity, the RA will ask the patient to describe the RIC procedure and whether study participation will have any effect on the medical care received. A patient with capacity to consent will be invited to participate. If the patient responds in the affirmative, this will be considered as verbal consent and the RA will instruct the paramedic to apply and start the autoRIC® device. This telephonic method to obtain consent in the field is based on a strategy developed and tested in prior prehospital emergency care trials [27, 28].

We anticipate ambulance transport times will be well under the 40-min RIC duration, so in most cases, the procedure will continue through ED arrival and part of the ED stay. When the ambulance crew arrives to the ED, providers in the ED will first assess the patient and provide usual care. The RA will collect the study case report form from the paramedic. Once initial care (e.g., provider assessment, repeat ECG, lab draws) is completed and the patient is placed under observation for further work-up, the RA will approach the participant, with approval from the treating physician, to complete the full informed consent process and describe additional data collection including medical record review and abstraction and collection and storage of blood samples for future testing. The RA will assess comprehension based on the patient’s ability to restate key study objectives and procedures and anticipated risks and benefits. If the patient consents, both RA and the participant will sign two copies of the informed consent form, one for study documentation and the other for the participant. If the patient refuses, he or she will be withdrawn from the study, and data collected up to that point, including completion status of RIC, will be de-identified and retained to address study objectives.

Participants may withdraw from the study at any point without consequence to their future care. If consent is withdrawn while RIC cycles are being administered, regardless of reason, the autoRIC® device will be stopped and removed from the arm. There are no anticipated risks to patients from abrupt termination of the RIC procedure. Care providers (i.e., paramedics or ED physicians) may terminate RIC if the patient becomes severely hypotensive (SBP < 90 mmHg), experiences an unsafe drop or rise in blood pressure, or for other safety reasons at the discretion of the provider. Discontinuation of RIC prematurely will be documented as the primary outcome. Specific reason(s) for discontinuation whether related to patient tolerability or safety will be documented to address secondary objectives and will be recorded for adverse event reporting.

### Data collection

**Fig. 4:**
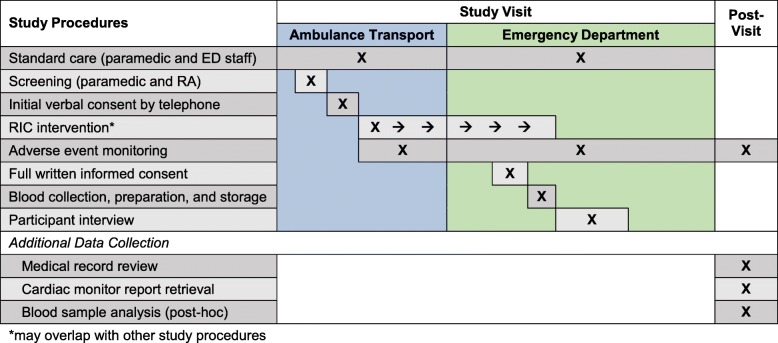
Timeline of study procedures and key protocol steps

The participant visit will be composed of segments of the ambulance transport and stay in the ED. Figure 4 illustrates the timeline of study procedures including data collected through direct patient involvement and following the participant’s study visit. Prior to the onset of screening and recruitment, study paramedics and research staff will undergo training on the study protocol and standard operating procedures.

During ambulance transport, the study paramedic will record information on a paper case report form (CRF). The CRF will be pre-populated with a unique participant identifier (ID). The paramedic will document general information, including incident date-time, patient name and contact information, and will also complete the screener. As previously described, eligible patients interested in learning more about the study will be connected to the RA by telephone. After verbal consent is obtained over the telephone, the paramedic will prepare the autoRIC® device (e.g., check battery, determine appropriate cuff size). The device will be placed on the patient and started according to the operator’s manual. During the procedure, the paramedic will record the time of RIC initiation and other key time points needed to compute secondary endpoints. While RIC is being administered, the paramedic will note observations and feedback on the screening and recruitment process, operation of the device, patient tolerability or adverse events, and any delays incurred due to RIC or other study procedures. If RIC is terminated prematurely, the paramedic will document the primary reason(s).

On arrival to the ED, the RA will meet the paramedic team and the participant. At this point, the paramedic will hand over the CRF to the RA and transfer study responsibilities, including safety and adverse event reporting. After initial ED care and while the patient is under observation and awaiting further work-up, the RA will complete the full informed consent process. This process may occur while the RIC procedure is still in progress. Although the study intervention will already be initiated, the participant will have the opportunity to decline participation in the in-person interview, access to medical records and other clinical data, and collection and storage of blood specimens for future testing. The RA will assess comprehension of consent by asking the patient to describe the purpose of the study, foreseeable risks and benefits of study participation, the possibility of unanticipated risks, and other elements of informed consent. No additional study procedures will occur until the consent process is complete and both the participant and RA sign necessary consent forms.

Once written consent is obtained, the RA will conduct a brief (10–15 min) semi-structured interview of the participant. Following an interview guide (see Additional file 1), the RA will begin with open-ended introductory questions on undergoing the RIC procedure (e.g., “what did it feel like?”, “how would you describe to someone else?”) and follow up with planned prompts and informal probing questions to elicit additional feedback. The RA will note participant responses in the CRF.

For future mechanistic studies of RIC, participants will be asked in a separate consent process for biospecimen collection and banking. A blood sample of 15 mL will be collected through existing venous access lines by clinical research staff trained in phlebotomy. When possible, this sample will be obtained with other blood draws taken as part of usual care. The blood for plasma will be collected using an EDTA tube (e.g., BD Vacutainer® plastic blood collection tube). Tubes will be labeled with the date and time of collection and the unique participant ID but no other identifiers. Specimens will be prepared according to standard procedures for separating plasma and will be stored in a − 80 °C freezer located in a locked area in the department’s research laboratory. No blood testing or analyses will be done under this protocol. In future studies, these samples may be used to investigate biomarkers and molecular pathways involved in the mechanism(s) of RIC.

Following the ED stay, the RA will enter data from the CRF (excluding participant name and contact information) into a secure, web-based database (Research Electronic Data Capture, REDCap). Additional study data will be collected by the RA via retrospective record review and abstraction. Relevant clinical data will be abstracted from the participant’s electronic medical record (e.g., medical history, diagnoses, lab results) and the EMS run sheet (e.g., time left scene, vital signs). Following each participant, the paramedic will also complete a brief questionnaire on screening, consent, and use of the autoRIC® device (see Additional file 2). Furthermore, automatic reports from the EMS provider’s LIFEPAK® 12 cardiac monitor will be retrieved and saved for potential additional information on vital signs and ECG patterns. No post-ED visits or interactions will be required of participants. Participants will, however, be contacted after 48 h, either by telephone or in-person if admitted, to assess for adverse events or other safety concerns related to the RIC intervention.

Since this is an open-label, low-risk pilot study at a single site, there will be no independent data and safety monitoring board. The principal investigator (MDP) will have access to all study data and ensure adequate data, and safety monitoring information is collected and reviewed in regularly scheduled meetings. During the enrollment, the focus of monitoring will be adverse event review and reporting, data security and participant privacy and confidentiality, and study conduct and data quality assurance. After all study data have been collected, a de-identified dataset will be stored in a secure location accessible only to the research staff for the purposes of analyzing data and summarizing results.

### Outcomes

The primary outcome measure will be the proportion of participants receiving 4 cycles of RIC without interruption using the autoRIC® device. We will also examine the total time (in minutes) and number of cycles that the RIC was administered. To address secondary objectives, the following quantitative measures will be computed: proportion of patients screened who are eligible (i.e., meet inclusion and exclusion criteria); proportion of patients recruited who agree to participate (i.e., verbal consent); timing (in minutes) of study procedures (i.e., arrival on scene, patient transport, screening, recruitment, RIC initiation); and proportion of participants who discontinue RIC due to discomfort or experience an anticipated adverse event. Qualitative data from paramedic-noted observations and semi-structured interviews of participants will be analyzed to generate themes on paramedic acceptability of the study protocol and participant experiences while undergoing RIC, respectively.

### Data analysis

Summary statistics (e.g., proportions, medians) will be used to characterize the study sample with respect to patient demographic and clinical characteristics and process factors related to the RIC procedure. The primary outcome measure will be the proportion of all participants receiving RIC who completed 4 cycles. The primary hypothesis will be evaluated by comparing the point estimate and its 95% CI to the predetermined benchmark of 0.80. Since this is a pilot study to collect feasibility data, no inferential statistical tests will be performed. Descriptive analyses will compare patient characteristics (demographics, medical history, clinical factors) and process measures (timing of study procedures) between RIC completers and non-completers.

Qualitative analysis of paramedic and participant responses will be performed with MAXQDA software (VERBI GmbH, Berlin, Germany). An iterative analysis strategy [29] used by other investigators will be employed in which a codebook is developed and refined based on a priori codes for expected responses and inductive codes as themes emerge during coding. All text will be recoded using a finalized codebook by the RA and other study personnel, and any discrepancies will be resolved by consensus. In mixed methods analysis combining qualitative and quantitative data [30], themes and concepts generated from textual coding will be related to demographic and clinical characteristics to investigate contributing factors to patient responses.

## Discussion

This pilot study will provide valuable evidence on the feasibility of administering RIC in the prehospital setting. Data on RIC intervention compliance (i.e., all 4 cycles completed) will inform the design and methods for a full-scale prehospital trial of RIC for acute STEMI. If this pilot study finds sub-optimal RIC completion (< 80%), we will identify strategies to maximize compliance during ground ambulance transport and incorporate into the final trial protocol. Further, based on any patient or process characteristics related to RIC discontinuation, appropriate statistical methods will be included in the data monitoring and analysis plan for the full-scale trial.

Previous clinical studies of RIC have shown that it is a safe procedure with minimal risk across various settings and patient populations. Botker et al. reported no local adverse effects, such as pain or thrombophlebitis, when RIC was administered in STEMI patients in the ambulance [19]. In two large clinical trials in patients undergoing CABG surgery, one observed no adverse events related to RIC [17]; the other found 5% of the RIC intervention group experienced skin petechiae, arm weakness, or altered sensation although none had long-term effects [16]. In a U.S. study of RIC during air medical transport of STEMI patients, only 2% of the patients stopped RIC due to discomfort [24]. Given the benign nature of this non-invasive procedure, no serious adverse events are expected. However, RIC may result in non-serious adverse events, such as minor arm discomfort, temporary discoloration of the arm or hand, and minor skin bruising or abrasions on the upper arm. Although these anticipated risks are not expected to have long-term consequences, they will need to be communicated to eligible patients in the informed consent process and to the public through community consultation. Our qualitative data on patient-reported experiences with RIC will provide key themes on which to focus in these methods.

Prehospital emergency research is a rapidly developing field with a growing public health imperative to improve clinical outcomes through high-quality research. However, optimal methods for conducting prehospital trials are not fully understood, especially for emergent, time-sensitive conditions like STEMI. Our secondary objectives to examine the timing of study procedures and obtain feedback from paramedics will contribute valuable insights for developing a logistically feasible and efficient trial protocol. Findings from this pilot study will also be relevant to other future prehospital trials of EMS interventions for acutely ill patients.

We acknowledge some limitations of this pilot study. First, although high-risk STEMI patients hold the most promise for benefiting from RIC, our feasibility aims will be addressed with a low-risk population. By using patients experiencing recent onset chest pain not suspected of STEMI, we will approximate a prehospital trial of RIC while allowing adequate time to consent and interview participants under observation in the ED. Moreover, a substantially shorter enrollment period will be required for non-STEMI patients. However, this study will not address all aspects of the feasibility of a trial in acute STEMI patients, particularly recruitment capability. Second, recruitment times will be restricted to daytime hours during the work week when RAs are available, and a substantial number of chest pain patients will be missed. We will retrospectively review EMS records to quantify the number missed. Third, we anticipate some ambulance transport times will be short (< 10 min), which may not be adequate to obtain verbal consent and start the RIC device. These occurrences will be documented and described. However, we expect most transport times to be 10–20 min and sufficient for completing the study procedures. Fourth, eligible patients are restricted to those able to speak English. Although this may exclude important segments of the population, English speakers were selected to allow for verbal consent and interviews by study personnel. Lastly, results from this single-site study may not represent the broader patient population and other EMS systems and providers.

Findings from this pilot study will inform the design and methods of a full-scale trial of prehospital RIC administered by EMS. To our knowledge, this will be the first study in the U.S. to assess the feasibility of RIC during ground ambulance transport. This work represents an essential initial step towards generating high-quality evidence on the clinical efficacy and safety of RIC. By addressing a major cause of morbidity and mortality following STEMI, this line of research has the potential for significant clinical and population health impact.

## Additional files

Additional file 1: Participant tolerability interview. (PDF 101 kb)

Additional file 2: Paramedic questionnaire. (PDF 41 kb)

## Abbreviations

CABG: Coronary artery bypass graft
CI: Confidence interval
CRF: Case report form
ECG: Electrocardiogram
ED: Emergency department
EMS: Emergency medical services
ID: Identifier
PCI: Percutaneous coronary intervention
RA: Research assistant
REDCap: Research Electronic Data Capture
RIC: Remote ischemic conditioning
SBP: Systolic blood pressure
STEMI: ST-elevation myocardial infarction
U.S.: United States
