# Supplementary material for: Feasibility of prehospital delivery of remote ischemic conditioning by emergency medical services in chest pain patients: protocol for a pilot study
Source: Pilot Feasibility Stud. 2019 Mar 13;5:42. doi: 10.1186/s40814-019-0431-8 (PMC6415490; doi:10.1186/s40814-019-0431-8)
Supplement: Supplementary file 1 — Participant tolerability interview. (PDF 101 kb) [file 40814_2019_431_MOESM1_ESM.pdf]

Participant Tolerability Interview (complete this section once RIC procedure is complete):

Time interview initiated: ______________

"I'm going to ask a few questions about your participation in the research study in which you've agreed to participate. During your ambulance ride, the paramedic placed a research device on your arm. It's called a RIC device and I'd like to ask you a few questions about it and what happened during your ride to the hospital as part of this study. Can we get started?"

"How does the arm on which you had the RIC device feel right now?"

____
____
____
____

"In your own words, please describe how it felt to have the RIC device on your arm."

____
____
____
____

"How would you describe what RIC is like to someone who has never undergone the procedure before?"

____
____
____
____

"I understand that you were experiencing chest pain symptoms prior to having the RIC device placed on your arm. After the RIC device was placed on your arm, did your symptoms change in any way? If so, how?"

____
____
____
____

"How would you rate the level of discomfort that RIC caused you on a scale from 0 (no discomfort at all) to 10 (the most discomfort you have ever experienced)? *Circle answer*

0 1 2 3 4 5 6 7 8 9 10

"For the following statements, please state whether you strongly agree, agree, are neutral, disagree, or strongly disagree:" *Circle answers*

1. "Having the RIC cuff on my arm was approximately what I expected it to be like after I received an explanation of RIC from the study coordinator over the telephone."

Strongly Agree	Agree	Neutral	Disagree	Strongly Disagree

2. "If I were having a heart attack, I would consider participating in a clinical trial to test the benefit of RIC by undergoing the entire 40-minute RIC procedure."

Strongly Agree	Agree	Neutral	Disagree	Strongly Disagree

3. "If I were having a heart attack, I would undergo the entire 40-minute RIC procedure if I knew it would benefit my health."

Strongly Agree	Agree	Neutral	Disagree	Strongly Disagree

Time interview completed: _______________

Signature of person completing form: ____Date:________________
