# Supplementary material for: Feasibility of prehospital delivery of remote ischemic conditioning by emergency medical services in chest pain patients: protocol for a pilot study
Source: Pilot Feasibility Stud. 2019 Mar 13;5:42. doi: 10.1186/s40814-019-0431-8 (PMC6415490; doi:10.1186/s40814-019-0431-8)
Supplement: Supplementary file 2 — Paramedic questionnaire. (PDF 41 kb) [file 40814_2019_431_MOESM2_ESM.pdf]

# Paramedic Questionnaire

Participant ID ______________________

## Paramedic Information:

Name ______________________

Email ______________________

Phone No. ______________________

Date of Interview ______________________

Age ______________________
(Age of paramedic)

Gender
◯ Male
◯ Female
◯ Other
◯ Do not wish to identify

How many years have you been a paramedic? ______________________

Have you ever participated in research before?
◯ Yes
◯ No

If so, briefly describe it? ______________________

In general, how do you feel about participating in clinical research as a paramedic?
◯ Very Positive
◯ Positive
◯ Neutral
◯ Negative
◯ Very Negative

Have you taken this survey for the RIC Pilot study before?
◯ Yes
◯ No

If yes, how many times? ______________________

**Paramedic Study Implementation Experience:**

**The following questions refer to your experience with patients who you screened for study participation for the UNC RIC Study. We are interested in your perspective and feedback. Please indicate you agreement with the questions below:**

| | |
|---|---|
| I had sufficient information and training to screen patients who met criteria for inclusion in the study | ◯ Strongly Agree<br>◯ Agree<br>◯ Neutral<br>◯ Disagree<br>◯ Strongly Disagree |
| Please explain: | __________________________________ |
| At any point during the screening process, was your ability to provide medical care affected? | ◯ Yes<br>◯ No |
| If yes, how was your ability to provide medical care affected? | __________________________________ |
| What feedback can you provide regarding the screening process? | __________________________________ |
| At any point during the consenting process (i.e calling the study coordinator) was your ability to provide medical care affected? | ◯ Yes<br>◯ No |
| If yes, how was your ability to provide medical care affected? | __________________________________ |
| What feedback can you provide regarding the consenting process if any? | __________________________________ |
| I had sufficient information and training to apply and start the RIC Device on the patient while providing normal care. | ◯ Strongly Agree<br>◯ Agree<br>◯ Neutral<br>◯ Disagree<br>◯ Strongly Disagree |
| Please explain why: | __________________________________ |
| At any point during the application and activation of the RIC device, was your ability to give medical care affected? | ◯ Yes<br>◯ No |
| If yes, please explain how application and activation of the RIC device changed the care you provided. | __________________________________ |

What feedback can you provide regarding the application and activation of the autoRIC device?

__________________________________

Overall, the training provided for the study adequately prepared you to screen, consent and use the RIC device.

- ◯ Strongly Agree
- ◯ Agree
- ◯ Neutral
- ◯ Disagree
- ◯ Strongly Disagree

Please explain why:

__________________________________

In your opinion, what are the biggest obstacles for future pre-hospital clinical trials involving the RIC device?

__________________________________

In your opinion, what are the biggest obstacles for future pre-hospital clinical trials involving paramedics?

__________________________________
